# (*R*)-(1-Ammonio­eth­yl)phospho­nate

**DOI:** 10.1107/S1600536810030308

**Published:** 2010-08-11

**Authors:** José A. Fernandes, Filipe A. Almeida Paz, Sérgio M. F. Vilela, João P. C. Tomé, José A. S. Cavaleiro, Paulo J. A. Ribeiro-Claro, João Rocha

**Affiliations:** a Department of Chemistry, University of Aveiro, CICECO, 3810-193 Aveiro, Portugal; b Department of Chemistry, University of Aveiro, QOPNA, 3810-193 Aveiro, Portugal

## Abstract

The title compound, C_2_H_8_NO_3_P, crystallizes in its zwitterionic form H_3_N^+^CH(CH_3_)PO(O^−^)(OH). In the crystal, the molecules are linked by N—H⋯O and O—H⋯O hydrogen bonds.

## Related literature

For the anti­bacterial activity of the title compound, see: Allen *et al.* (1979 ▶). For the use of the title compound as a co-crystallizing inhibitor on the X-ray structure of the alanine racemase from *Bacillus anthracis*, a potential anti-anthrax drug target, see: Au *et al.* (2008 ▶). For examples of coordination compounds of the title compound, see: Cui *et al.* (2006 ▶); Carraro *et al.* (2008 ▶). For a description of the graph-set notation for hydrogen-bonded aggregates, see: Grell *et al.* (1999 ▶). For previous work from our research group on the assembly of coordination polymers using phospho­nic-based mol­ecules, see: Cunha-Silva, Ananias *et al.* (2009 ▶); Cunha-Silva, Lima *et al.* (2009 ▶); Shi, Cunha-Silva *et al.* (2008 ▶); Shi, Trindade *et al.* (2008 ▶).
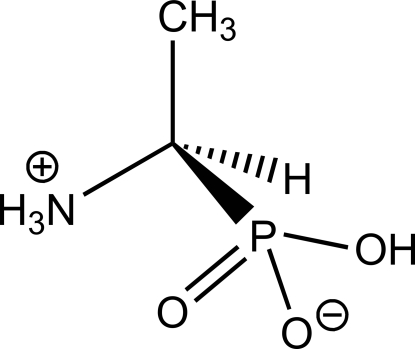


## Experimental

### 

#### Crystal data

C_2_H_8_NO_3_P
*M* *_r_* = 125.06
Orthorhombic, 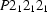

*a* = 4.8256 (1) Å
*b* = 10.3928 (3) Å
*c* = 10.4668 (3) Å
*V* = 524.93 (2) Å^3^
*Z* = 4
Mo *K*α radiation
μ = 0.42 mm^−1^
*T* = 150 K
0.12 × 0.08 × 0.04 mm

#### Data collection

Bruker X8 Kappa CCD APEXII diffractometer
Absorption correction: multi-scan (*SADABS*; Sheldrick, 1997 ▶) *T*_min_ = 0.951, *T*_max_ = 0.983
7972 measured reflections
2535 independent reflections
2362 reflections with *I* > 2σ(*I*)
*R*_int_ = 0.027

#### Refinement

*R*[*F* ^2^ > 2σ(*F* ^2^)] = 0.025
*wR*(*F* ^2^) = 0.070
*S* = 1.07
2535 reflections
77 parameters
7 restraints
H atoms treated by a mixture of independent and constrained refinement
Δρ_max_ = 0.50 e Å^−3^
Δρ_min_ = −0.26 e Å^−3^
Absolute structure: Flack (1983 ▶), 1051 Friedel pairs
Flack parameter: 0.00 (8)

### 

Data collection: *APEX2* (Bruker, 2006 ▶); cell refinement: *SAINT-Plus* (Bruker, 2006 ▶); data reduction: *SAINT-Plus*; program(s) used to solve structure: *SHELXTL* (Sheldrick, 2008 ▶); program(s) used to refine structure: *SHELXTL*; molecular graphics: *DIAMOND* (Brandenburg, 2009 ▶); software used to prepare material for publication: *SHELXTL*.

## Supplementary Material

Crystal structure: contains datablocks global, I. DOI: 10.1107/S1600536810030308/tk2694sup1.cif

Structure factors: contains datablocks I. DOI: 10.1107/S1600536810030308/tk2694Isup2.hkl

Additional supplementary materials:  crystallographic information; 3D view; checkCIF report

## Figures and Tables

**Table 1:** Hydrogen-bond geometry (Å, °)

*D*—H⋯*A*	*D*—H	H⋯*A*	*D*⋯*A*	*D*—H⋯*A*
O2—H4⋯O3^i^	0.92 (1)	1.63 (1)	2.5484 (10)	175 (2)
N1—H1⋯O1^ii^	0.94 (1)	1.91 (1)	2.8033 (11)	157 (1)
N1—H2⋯O3^iii^	0.94 (1)	1.90 (1)	2.8369 (12)	178 (1)
N1—H3⋯O1^iv^	0.95 (1)	1.87 (1)	2.8160 (12)	171 (1)

Symmetry codes: (i) 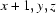; (ii) 
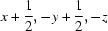
; (iii) 
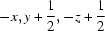
; (iv) 
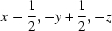
.

**Fig. 1.**
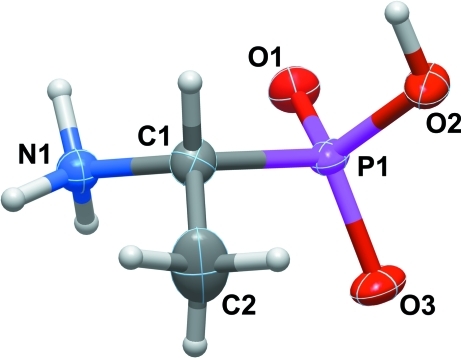
Molecular structure of the title compound. Displacement ellipsoids are drawn at the 80% probability level and the atomic labeling is provided for all non-hydrogen atoms.

**Fig. 2.**
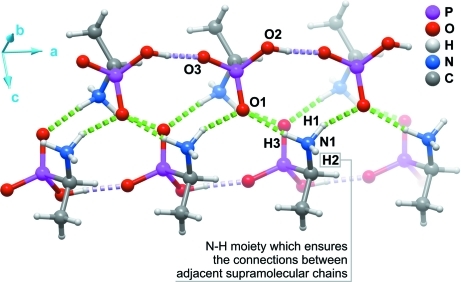
Supramolecular one-dimensional zigzag chain running parallel to the [100] direction of the unit cell. The inner N—H···O interactions composing the graph set motif C12(4) are represented as dashed green lines, while the outer O—H···O bonds as violet dashed green lines. For hydrogen bonding geometrical details see Table 1. Symmetry operations used to generate equivalent atoms have been omitted for simplicity.

**Fig. 3.**
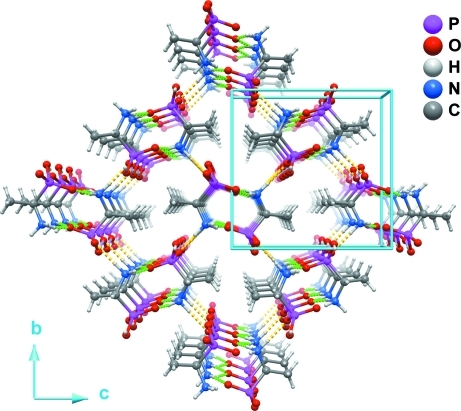
Crystal packing of the title compound viewed in perspective along the [100] direction of the unit cell. Hydrogen bonds are represented as dashed green (intra-chain N—H···O type), violet (O—H···O) or orange (inter-chain N—H···O interactions) lines.

## supplementary crystallographic information

### Comment

The title compound, *R*-1-aminoethylphosphonic acid (C_2_H_8_NO_3_P), is the phosphonic analogue of the amino acid alanine and, therefore, it is commonly represented as *L*-Ala-P (Au *et al.*, 2008). It presents antibacterial activity (Allen *et al.*, 1979) and it has been employed as inhibitor in the crystallization of the enzyme alanine racemase from *Bacillus anthracis* (Au *et al.*, 2008). Remarkably, only two coordination compounds containing *L*-Ala-P as ligand are known, namely a racemic coordination polymer of zinc (Cui *et al.*, 2006) and a chiral molybdenum cluster (Carraro *et al.*, 2008). Following our interest in the use of phosphonic acid molecules in the construction of multi-dimensional coordination polymers (Cunha-Silva, Ananias *et al.*, 2009; Cunha-Silva, Lima *et al.*, 2009; Shi, Cunha-Silva *et al.*, 2008; Shi, Trindade *et al.*, 2008), herein we wish to describe the crystal structure of the title compound.

The title compound crystallises in its zwitterionic form in which the acidic phosphonate moiety donates one proton to the amino group (Fig. 1). Individual molecular units are disposed in a zigzag fashion along the [100] direction of the unit cell, leading to the formation of a supramolecular chain held together by a combination of the inner ···O1···H1—N1^+^—H3··· hydrogen bonds [graph set motif C^1^_2_(4), Grell *et al.* (1999) - green dashed bonds in Fig. 2], and the outer isolated O2—H4···O3 interactions (violet dashed bonds in Fig. 2). Supramolecular chains are in turn interconnected in the *bc* plane *via* the remnant N1^+^—H2···O2 hydrogen bonds as depicted in Fig. 3 (orange dashed lines). It is noteworthy that all hydrogen bonding interactions are rather strong and directional: while the internuclear D···A distances range from 2.5484 (10) to 2.8369 (12) Å, the 〈(DHA) are all greater than 157°. As depicted in Fig. 3, the crystal packing promotes a close proximity between the substituent —CH_3_ groups which point toward each other.

### Experimental

The title compound was purchased from Sigma-Aldrich (>97.0%, Fluka) and was used as received without purification. Suitable single crystals were grown from an aqueous solution over a period of two weeks.

^1^H-NMR (300.13 MHz, D_2_O) δ: 1.27 (dd, 3H, *J*(^1^H-^1^H) = 7.3 Hz and *J*(^1^H-^31^P)= 14.8 Hz, CH_3_) and 3.19 (dq, 1H, *J*(^1^H-^1^H)= 7.3 Hz and *J*(^1^H-^31^P) = 12.7 Hz, CH).

^13^C-NMR (75.47 MHz, D_2_O) δ: 16.4 (d, *J*(^13^C-^31^P) = 2.6 Hz,CH_3_) and 47.6 (d, *J*(^13^C-^31^P) = 145.1 Hz,CH).

^31^P-NMR (121.49 MHz, D_2_O) δ: 14.8 (dq, *J*(^31^P-^1^H) = 13.8 and 14.6 Hz).

### Refinement

Hydrogen atoms bound to carbon were located at their idealized positions and were included in the final model in the riding-motion approximation with C—H = 1.00 Å (tertiary C—H) or 0.98 Å (–CH_3_). The isotropic thermal displacement parameters for these atoms were fixed at 1.2 (for methine-H) or 1.5 (methyl-H) times *U*_eq_ of the respective parent atom.

Hydrogen atoms associated with the protonated —NH_3_^+^ group or the pendant —OH moiety were located from difference Fourier maps and were included in the final model with the distances restrained to 0.95 (1) Å and *U*_iso_*=1.5×U*_eq_ of the respective parent atom. The H···H distances of the —NH_3_^+^ terminal group were further restrained to 1.55 (1) Å in order to ensure a chemically reasonable geometry for this moiety.

### Crystal data

**Table:**

C_2_H_8_NO_3_P	*F*(000) = 264
*M**_r_* = 125.06	*D*_x_ = 1.583 Mg m^−^^3^
Orthorhombic, *P*2_1_2_1_2_1_	Mo *K*α radiation, λ = 0.71073 Å
Hall symbol: P 2ac 2ab	Cell parameters from 3869 reflections
*a* = 4.8256 (1) Å	θ = 2.8–35.9°
*b* = 10.3928 (3) Å	µ = 0.42 mm^−^^1^
*c* = 10.4668 (3) Å	*T* = 150 K
*V* = 524.93 (2) Å^3^	Prism, colourless
*Z* = 4	0.12 × 0.08 × 0.04 mm

### Data collection

**Table:**

Bruker X8 Kappa CCD APEXII diffractometer	2535 independent reflections
Radiation source: fine-focus sealed tube	2362 reflections with *I* > 2σ(*I*)
graphite	*R*_int_ = 0.027
ω and phi scans	θ_max_ = 36.3°, θ_min_ = 3.9°
Absorption correction: multi-scan (*SADABS*; Sheldrick, 1997)	*h* = −7→8
*T*_min_ = 0.951, *T*_max_ = 0.983	*k* = −16→17
7972 measured reflections	*l* = −17→16

### Refinement

**Table:**

Refinement on *F*^2^	Secondary atom site location: difference Fourier map
Least-squares matrix: full	Hydrogen site location: inferred from neighbouring sites
*R*[*F*^2^ > 2σ(*F*^2^)] = 0.025	H atoms treated by a mixture of independent and constrained refinement
*wR*(*F*^2^) = 0.070	*w* = 1/[σ^2^(*F*_o_^2^) + (0.0415*P*)^2^] where *P* = (*F*_o_^2^ + 2*F*_c_^2^)/3
*S* = 1.07	(Δ/σ)_max_ < 0.001
2535 reflections	Δρ_max_ = 0.50 e Å^−^^3^
77 parameters	Δρ_min_ = −0.26 e Å^−^^3^
7 restraints	Absolute structure: Flack (1983), 1051 Friedel pairs
Primary atom site location: structure-invariant direct methods	Flack parameter: 0.00 (8)

### Special details

**Table:**

Geometry. All e.s.d.'s (except the e.s.d. in the dihedral angle between two l.s. planes) are estimated using the full covariance matrix. The cell e.s.d.'s are taken into account individually in the estimation of e.s.d.'s in distances, angles and torsion angles; correlations between e.s.d.'s in cell parameters are only used when they are defined by crystal symmetry. An approximate (isotropic) treatment of cell e.s.d.'s is used for estimating e.s.d.'s involving l.s. planes.
Refinement. Refinement of *F*^2^ against ALL reflections. The weighted *R*-factor *wR* and goodness of fit *S* are based on *F*^2^, conventional *R*-factors *R* are based on *F*, with *F* set to zero for negative *F*^2^. The threshold expression of *F*^2^ > σ(*F*^2^) is used only for calculating *R*-factors(gt) *etc*. and is not relevant to the choice of reflections for refinement. *R*-factors based on *F*^2^ are statistically about twice as large as those based on *F*, and *R*- factors based on ALL data will be even larger.

### Fractional atomic coordinates and isotropic or equivalent isotropic displacement parameters (Å^2^)

**Table:**

	*x*	*y*	*z*	*U*_iso_*/*U*_eq_	
P1	0.12299 (5)	0.08570 (2)	0.11153 (2)	0.01023 (6)	
O1	0.15178 (17)	0.13133 (7)	−0.02404 (7)	0.01436 (13)	
O2	0.33072 (15)	−0.02579 (7)	0.14607 (8)	0.01551 (14)	
H4	0.512 (2)	0.0009 (18)	0.1437 (15)	0.023*	
O3	−0.16015 (15)	0.03922 (8)	0.15035 (8)	0.01671 (15)	
N1	0.15332 (19)	0.34471 (8)	0.15951 (8)	0.01264 (14)	
H1	0.290 (2)	0.3648 (15)	0.0983 (11)	0.019*	
H2	0.152 (3)	0.4080 (12)	0.2239 (10)	0.019*	
H3	−0.0211 (19)	0.3439 (15)	0.1171 (13)	0.019*	
C1	0.2286 (2)	0.21731 (10)	0.21766 (9)	0.01326 (17)	
H1A	0.4352	0.2143	0.2248	0.016*	
C2	0.1111 (4)	0.20387 (11)	0.35212 (10)	0.0253 (2)	
H2A	0.1924	0.2698	0.4075	0.038*	
H2B	0.1557	0.1184	0.3858	0.038*	
H2C	−0.0906	0.2148	0.3495	0.038*	

### Atomic displacement parameters (Å^2^)

**Table:**

	*U*^11^	*U*^22^	*U*^33^	*U*^12^	*U*^13^	*U*^23^
P1	0.00725 (9)	0.01088 (9)	0.01254 (9)	−0.00054 (8)	−0.00037 (8)	0.00118 (8)
O1	0.0141 (3)	0.0168 (3)	0.0121 (3)	−0.0005 (3)	−0.0003 (3)	0.0012 (2)
O2	0.0087 (3)	0.0130 (3)	0.0248 (4)	0.0004 (2)	−0.0024 (2)	0.0025 (3)
O3	0.0076 (3)	0.0195 (3)	0.0229 (3)	−0.0017 (2)	0.0002 (3)	0.0051 (3)
N1	0.0126 (3)	0.0122 (3)	0.0132 (3)	0.0000 (3)	0.0000 (3)	−0.0004 (3)
C1	0.0134 (4)	0.0137 (4)	0.0126 (4)	−0.0001 (3)	−0.0025 (3)	0.0011 (3)
C2	0.0425 (7)	0.0216 (5)	0.0118 (4)	0.0001 (5)	0.0004 (5)	0.0019 (4)

### Geometric parameters (Å, °)

**Table:**

P1—O1	1.5025 (8)	N1—H2	0.942 (7)
P1—O3	1.5051 (8)	N1—H3	0.951 (7)
P1—O2	1.5742 (8)	C1—C2	1.5238 (16)
P1—C1	1.8344 (10)	C1—H1A	1.0000
O2—H4	0.918 (9)	C2—H2A	0.9800
N1—C1	1.5018 (13)	C2—H2B	0.9800
N1—H1	0.943 (8)	C2—H2C	0.9800
			
O1—P1—O3	116.12 (4)	N1—C1—C2	111.41 (9)
O1—P1—O2	112.98 (4)	N1—C1—P1	110.17 (6)
O3—P1—O2	106.25 (4)	C2—C1—P1	112.80 (8)
O1—P1—C1	108.11 (5)	N1—C1—H1A	107.4
O3—P1—C1	109.15 (5)	C2—C1—H1A	107.4
O2—P1—C1	103.46 (4)	P1—C1—H1A	107.4
P1—O2—H4	112.2 (12)	C1—C2—H2A	109.5
C1—N1—H1	107.5 (10)	C1—C2—H2B	109.5
C1—N1—H2	109.1 (9)	H2A—C2—H2B	109.5
H1—N1—H2	109.6 (9)	C1—C2—H2C	109.5
C1—N1—H3	113.3 (10)	H2A—C2—H2C	109.5
H1—N1—H3	107.7 (9)	H2B—C2—H2C	109.5
H2—N1—H3	109.5 (10)		
			
O1—P1—C1—N1	33.30 (8)	O1—P1—C1—C2	158.48 (8)
O3—P1—C1—N1	−93.86 (7)	O3—P1—C1—C2	31.32 (10)
O2—P1—C1—N1	153.33 (7)	O2—P1—C1—C2	−81.49 (9)

### Hydrogen-bond geometry (Å, °)

**Table:**

*D*—H···*A*	*D*—H	H···*A*	*D*···*A*	*D*—H···*A*
O2—H4···O3^i^	0.92 (1)	1.63 (1)	2.5484 (10)	175.(2)
N1—H1···O1^ii^	0.94 (1)	1.91 (1)	2.8033 (11)	157.(1)
N1—H2···O3^iii^	0.94 (1)	1.90 (1)	2.8369 (12)	178.(1)
N1—H3···O1^iv^	0.95 (1)	1.87 (1)	2.8160 (12)	171.(1)

Symmetry codes: (i) *x*+1, *y*, *z*; (ii) *x*+1/2, −*y*+1/2, −*z*; (iii) −*x*, *y*+1/2, −*z*+1/2; (iv) *x*−1/2, −*y*+1/2, −*z*.
